# Hepatic segmental atrophy: a diagnostic challenge with variable clinicopathologic features and an association with cardiovascular disease

**DOI:** 10.1007/s00428-025-04094-6

**Published:** 2025-04-15

**Authors:** Negin Farsi, Monica Sanchez-Avila, Melissa Duarte, Domenika Ortiz Requena, Turky Alkathery, Nemencio R. Ronquillo, Monica Garcia-Buitrago, Lisa M. Stoll, Elizabeth A. Montgomery, Kathleen Byrnes

**Affiliations:** 1 Department of Pathology and Laboratory Medicine, Miller School of Medicine, University of Miami, Miami, FL 33136 USA; 2 Department of Pathology and Laboratory Medicine, Luke University Health Network, Bethlehem, StPA 18015 USA; 3 Department of Pathology and Immunology, Washington University School of Medicine, St. Louis, MO 63310 USA; 4 Department of pathology, University of Tennessee Health Science Center, Memphis, TN 38163 USA

**Keywords:** Liver, Segmental atrophy, Cardiovascular disease, Elastosis

## Abstract

Hepatic segmental atrophy is an underrecognized pseudotumor that can cause diagnostic challenges. These lesions can be mass-forming and demonstrate a range of pathologic features that can lead to diagnostic errors on both imaging studies, on frozen section, and in biopsy material. The aim of this study was to better understand the clinicopathologic features of this condition as well as its association with cardiovascular disease. A retrospective computerized search of the files of two institutions was conducted spanning 2012 to 2024. All surgical slides were reviewed, and clinical and demographic information was collected. There were 45 patients, including 23 men and 22 women with median age of 63 years. Most (35/45, 78%) patients had a history of hypertension or cardiovascular disease. Thirty patients had a history of malignant neoplasm, and seven had cirrhosis. Histologically, the cases showed variable histologic features, ranging from nodular elastosis to more subtle lesions featuring degenerative hepatocytes with relative preservation of bile ducts between them. Association of these lesions with remote vascular injury might be explained by cardiovascular disease, most commonly hypertension. The current case series emphasizes the importance of recognizing this lesion and its association with cardiovascular diseases. While lobular or segmental atrophy of the liver has been recognized as a complication of many benign and malignant conditions, pathologists should be aware that this can present as a mass lesion.

## Introduction

**Fig. 1:**
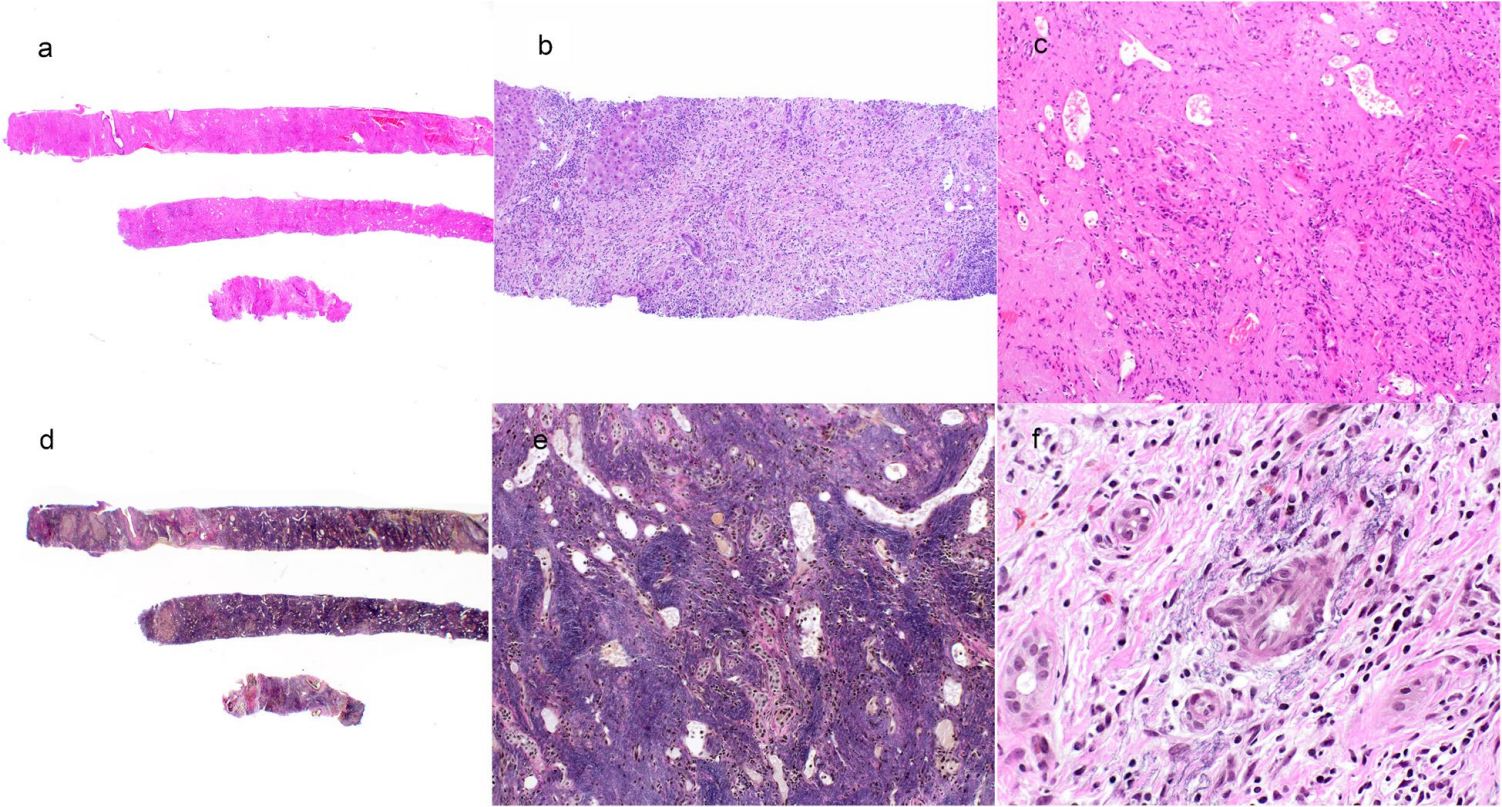
Liver biopsy showing segmental atrophy, loss of hepatocytes. **a** Hematoxylin and eosin scale bar: 200 µm;** b** hematoxylin and eosin scale bar: 100 µm, **c** hematoxylin and eosin scale bar: 50 µm, nodular elastosis (**d** elastic stain scale bar: 200 µm*, bottom middle*:** e** elastic stain scale bar: 50 µm) and thick arteries (**f** elastic stain, scale bar: 20 µm)

**Fig. 2:**
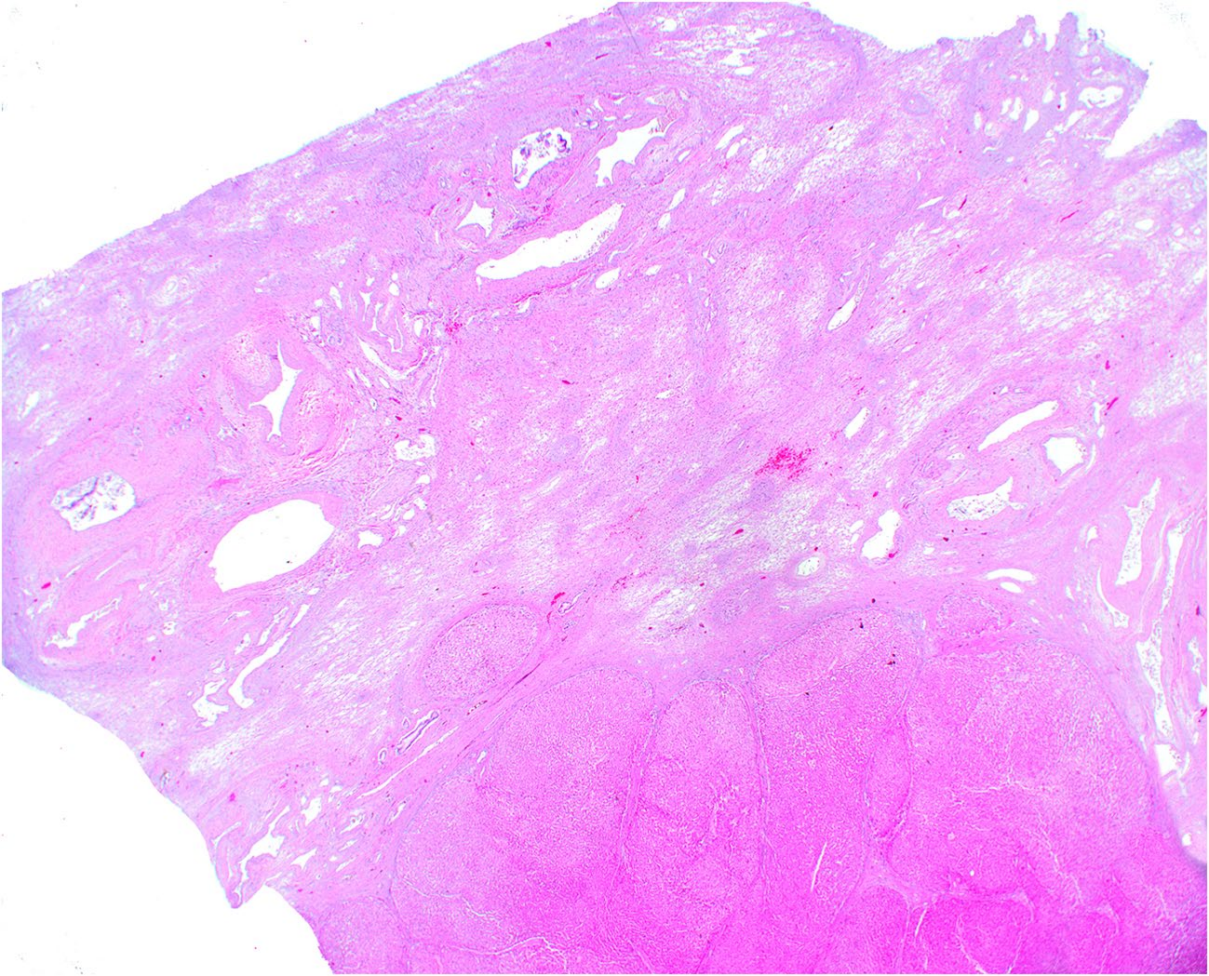
Wedge resection showing segmental atrophy

Lobar and segmental atrophy of the liver (SAL) has been recognized with the routine use of computed tomography scans and ultrasound [1]. Segmental atrophy of liver is a rare condition that was first described by Singhi et al. [2] as a pseudotumor of liver manifesting as varying stages of the lesion. The pathogenesis of this lesion remains incompletely understood, but remote vascular injury and benign and malignant lesions of the liver or bile ducts might be contributing factors. Some of the described pathologic features have included the presence of abnormally thickened blood vessels throughout the lesion, involving both arteries and veins. Vascular thrombosis, fibrosis, and recanalization have all been described. The compromised perfusion of the hepatic parenchyma leads to parenchymal atrophy and loss of hepatocytes—essentially infarction over time. Early lesions are composed of collapsed hepatocytes interspersed with normal hepatocytes and bile ductular reaction with mild to moderate elastotic changes in the background. More advanced and older lesions are characterized by the presence of more elastosis, biliary cysts, and absence of bile ductular proliferation, which eventually evolves to nodular elastosis, which consists of small islands of hepatocytes within an elastic rich matrix (Figs. 1 and 2). Ruptured biliary cysts can create focal fibrosis and granulation tissue. There have been a few additional case reports and series describing the histopathologic and clinical presentations of this lesion following the initial description by Singhi et al. [2], but lack of awareness and clinicopathologic overlap of this mass-forming lesion with other hepatic lesions can create diagnostic challenges for clinicians, radiologists, and pathologists alike. A study from 2020 published in abstract form suggested an association between hepatic segmental atrophy and long-standing cardiovascular disease [3]. The aim of this study was to review the clinicopathologic features of hepatic segmental atrophy based on a large cohort of patients.

## Methods

Following institutional review board approval, we searched for “segmental atrophy” and “liver” from the pathology files of three hospitals (Washington University in St. Louis, Jackson Memorial hospital, and University of Miami) for in-house and consult cases reviewed between 2012 and 2024. A subset of cases that were encountered prospectively in the course of daily patient care were added. All cases were subsequently assessed for histologic features of hepatic segmental atrophy as previously described by Singhi et al. [2]. Cases without all of the diagnostic features were excluded, including one case that had granulomatous inflammation, two cases that were necrotic as a result of chemotherapy, and two cases that were hemangiomas. Two cases were for the same patient who had a biopsy and a subsequent wedge resection. The wedge resections were included for the evaluation. All available immunohistochemical and histochemical stains were examined. Demographic and clinical history for each case were collected by reviewing the electronic medical records. When clinical and demographic information for consultation cases were unavailable, the original institution was contacted for such information.

## Results

There were 45 cases in total from two different institution, including 19 core biopsies, 18 wedge resections, four partial hepatectomies, and four liver explants. There were 23 men and 22 women with an average age of 60.6 years and median of 63 years (range: 6 weeks old to 81 years).

Imaging studies (CT scan or MRI) were available for 37/45 (82%) cases, all of which demonstrated a mass-forming lesions suspicious for neoplasm. For those cases for which imaging studies were not available, the lesion was identified incidentally during surgery, or the imaging findings were not provided by the original institution for consult cases. The lesion was reported as subcapsular in only 13 of 45 (28%) patients on the imaging studies. The average size of the lesions was 1.72 cm (range 0.5 to 7.5 cm). The lesions arose in the right lobe (22/45 patients) and left lobe (19/45), including 11 cases in segment 4, five cases in segment 3, four cases in segment 8, one case in segment 7, two cases in segment 6, and four cases in segment 5. One case showed atrophy in segment 8, 4, and 5.

The histologic features of the lesion included elastosis and thick blood vessels in all but one of the cases (possibly a reflection of sampling error). Collapsed hepatic parenchyma with occasional small islands of atrophic hepatocytes was recognized in 44 of 45 cases. Ductular proliferation/ductular reaction was identified in 11 of 45 cases (24%). There were three cases with von-Meyenberg complexes. None of the cases showed findings of focal nodular hyperplasia or porto-sinusoidal vascular disease.

Most (35/45,78%) patients had a history of hypertension, and four patients had both atrial fibrillation and hypertension (Table 1). Two patients had a history of extensive cardiovascular disease, one of whom had a history of myocardial infarction requiring stenting, complicated by a cerebrovascular accident. Two patients only had diabetes mellitus, and six patients had both diabetes mellitus and hypertension. Seven patients had cirrhosis (three HCV associated and two NASH induced). Thirty of the patients had a history of a malignant neoplasm (30 patients), including cholangiocarcinoma/gallbladder adenocarcinoma (4), hepatocellular carcinoma (3), gastroesophageal adenocarcinoma (1), gastric adenocarcinoma (2), colon adenocarcinoma (5), pancreatic adenocarcinoma (4), lung small cell carcinoma (1), invasive ductal carcinoma of breast (1), colon neuroendocrine tumor (1), neuroendocrine carcinoma (1), papillary renal cell carcinoma (1), neuroendocrine tumor of pancreas (1), and prostate adenocarcinoma (1), liposarcoma (1). One patient had a history of both hepatocellular carcinoma and cholangiocarcinoma. The pediatric case was a 6-week-old with a history of metastatic neuroblastoma and received chemotherapy and total parenteral nutrition. None of the patients (0/45) showed any evidence of cancer in the biopsies or wedge resections. One patient had a prior history of metastatic colon cancer to the liver, but the biopsy was not near the site of metastasis. The remaining cases did not have any metastatic disease present in the liver at the time of the procedure. There was one biopsy in a patient with hepatocellular carcinoma and one explant with cholangiocarcinoma, but the tumor was remote from the areas of segmental atrophy.

**Table 1:** Clinical characteristics of patients with segmental atrophy of liver

Type of specimen	Size (average)	Gender	History of cancer	History of cardiovascular disease
Biopsy: 19	1.72 cm	Female: 22	Yes: 30	Hypertension: 35
Partial hepatectomy: 4		Male: 23	No: 15	Atrial fibrillation: 4
Wedge: 18				Diabetes mellitus: 7
Explant: 4				
Total number of cases: 45				

Of the nine patients without hypertension or cardiovascular disease, two had only DM and a history of carcinoma (colonic adenocarcinoma and gastroesophageal adenocarcinoma). Six had a prior history of malignancy including colonic adenocarcinoma (*n* = 1), small cell carcinoma of the lung (*n* = 1), neuroblastoma (*n* = 1), hepatocellular carcinoma, and cholangiocarcinoma (*n* = 2).Of these six patients, two were previously treated with chemotherapy. The remaining patient had a history of alcohol abuse disorder but no evidence of steatohepatitis in the background of the core biopsy.

The detailed information on clinical features of each patient in our including size of the lesion, specimen type, presence or absence and type of cardiovascular disease, presence or absence of DM, and clinical history of cancer and/or chemotherapy are demonstrated in Table 2.

**Table 2:** Detailed clinical features of study patient population

Case #	Age	Gender	Specimen type	Location	Liver lobe	Size (cm)	Fibrosis	CVD	Type of CVD	Hx of malignancy	Hx of CXR
1	81	M	Partial hepatectomy	Segment 3	Left	2.5	None	Yes	HTN	None	None
2	59	F	Core biopsy	Segment 4	Left	0.5	-	Yes	HTN	None	None
3	62	F	Core biopsy	Segment 8	Right	1.4	Cirrhosis	Yes	HTN, previous MI	None	None
4	67	M	Core biopsy	Segment 8	Right	4.3	Cirrhosis	Yes	HTN	None	None
5	70	M	Core biopsy	Segment 7	Right	1.9	None	Yes	HTN	Prostate adenocarcinoma	None
6	59	M	Wedge biopsy	Segment 3	Left	1.6	None	Yes	HTN, AF	None	None
7	65	M	Explant	Subcapsular	Right	5	Bridging	Yes	HTN	Cholangiocarcinoma	Yes
8	63	M	Wedge biopsy	Unknown	-	1	None	Yes	HTN	Gastric adenocarcinoma	Yes
9	27	M	Core biopsy	Right lobe	Right	0.5	None	None	None	None	None
10	49	F	Explant	Segment 4	Left	1	Periportal	Yes	HTN	Cholangiocarcinoma	Yes
11	68	M	Core biopsy	Segment 5	Right	0.6	Portal	Yes	HTN	HCC	None
12	47	F	Core biopsy	Subcapsular	Left	1.5	None	Yes	HTN	None	None
13	37	F	Wedge biopsy	Segment 3	Left	0.3	-	Yes	HTN, DM	panNET	None
14	61	M	Wedge biopsy	Segment 4	Left	1	-	Yes	DM	GE adenocarcinoma	Yes
15	63	F	Partial hepatectomy	Segment 6 (subcapsular)	Right	1.5	None	Yes	HTN	Colonic adenocarcinoma	Yes
16	71	F	Wedge biopsy	Segment 4 (subcapsular)	Left	0.9	None	Yes	HTN, DM	Pancreatic adenocarcinoma	None
17	75	F	Core biopsy	Unknown	-	1	-	Yes	HTN	Liposarcoma	None
18	62	M	Core biopsy	Left lateral	Left	0.4	-	Yes	HTN	Pancreatic adenocarcinoma	Yes
19	78	M	Wedge biopsy	Subcapsular	Left	2.1	-	Yes	HTN	Esophageal adenocarcinoma	Yes
20	75	M	Wedge biopsy	Segment 3	Left	1	-	Yes	HTN, DM	None	None
21	58	M	Wedge biopsy	Segment 4	Left	2	Portal	None	None	Colonic adenocarcinoma	None
22	51	F	Wedge biopsy	Unknown	-	0.4	-	None	None	Small cell carcinoma of lung	Yes
23	58	F	Explant	Right lobe	Right	7.5	Cirrhosis	Yes	HTN	None	None
24	68	F	Wedge biopsy	Unknown	-	0.4	None	None	None	Pancreatic adenocarcinoma	None
25	62	M	Core biopsy	Segment 7	Right	4.2	None	Yes	HTN, DM	None	None
26	67	M	Wedge biopsy	Segment 4	Left	3	None	Yes	HTN, DM	Papillary renal cell carcinoma	None
27	75	F	Wedge biopsy	Segment 3	Left	1.6	None	Yes	HTN	NEC	Yes
28	76	F	Wedge biopsy	Right lobe	Right	2	None	Yes	HTN	Colonic adenocarcinoma	None
29	73	F	Partial hepatectomy	Right lobe	Right	2.7	None	Yes	HTN	Colonic adenocarcinoma	Yes
30	72	M	Core biopsy	Segment 4a	Left	2.9	Cirrhosis	Yes	HTN, DM	cholangiocarcinoma	Yes
31	63	F	Core biopsy	Segment 8	Right	1.4	None	Yes	HTN	Gastric adenocarcinoma	Yes
32	40	F	Core biopsy	Subcapsular	Right	0.5	None	Yes	HTN	Invasive ductal carcinoma	Yes
33	36	M	Segmental resection	Segment 5 (subcapsular)	Right	1.8	Portal	Yes	DM	Colonic adenocarcinoma	Yes
34	55	M	Core biopsy	Segment 5/6 (subcapsular)	Right	2.1	Cirrhosis	Yes	HTN	None	-
35	77	M	Wedge biopsy	Subcapsular	Right	0.5	Portal	Yes	HTN, AF	None	-
36	6 weeks	F	Core biopsy	Segment 4 (subcapsular)	Left	-	None	None	None	Neuroblastoma	-
37	70	M	Wedge biopsy	Superior right lobe (subcapsular)	Right	1	None	Yes	HTN, AF	Esophageal adenocarcinoma	Yes
38	63	M	Core biopsy	Left lobe	Left	-	None	Yes	Cardiovascular disease	None	-
39	53	M	Core biopsy	Right lobe	Right	1.9	Periportal	Yes	HTN	None	-
40	69	F	Wedge resection	Segment 4 and 5	Right	1.1	-	Yes	HTN, AF	Cholangiocarcinoma	Yes
41	76	F	Core biopsy	Segment 8 (subcapsular)	Right	1.2	None	Yes	HTN	Cecal NET	-
42	57	F	Native liver	Right lobe	Right	-	Cirrhosis	None	None	HCC, cholangiocarcinoma	Yes
43	63	F	Wedge resection	Segment 4	Left	2	Portal	Yes	HTN	None	
44	58	M	Core biopsy	Segment 8, 4a, and 5 (subcapsular)	Right	-	Cirrhosis	None	None	HCC	
45	52	F	Wedge biopsy	Segment 4b	Left	0.6	Portal	Yes	HTN	Pancreatic adenocarcinoma	Yes

*CVD* Cardiovascular disease, *DM* Diabetes mellitus, *HTN* Hypertension, *HCC* Hepatocellular carcinoma, *PanNET* Pancreatic neuroendocrine tumor, *NET* Neuroendocrine tumor, *NEC* Neuroendocrine carcinoma

## Discussion

Segmental atrophy of liver (SAL) is a rare and under-recognized pseudotumor of liver that results in diagnostic challenges for clinicians. These lesions are classically described as mass-forming and subcapsular, and the pathogenesis of this lesion is not entirely clear. Studies have speculated that remote vascular injury or vascular injury accompanying benign and malignant neoplasms is the key initiating factor [4, 5].

Hepatic atrophy can accompany many underlying liver diseases, including cirrhosis, cholangiocarcinoma, hepatic cysts, hepatocellular carcinoma, and hepatic failure [6]. Variable histologic features of this lesion, although distinct, can lead to erroneous diagnosis by the pathologist. The histological features range from parenchymal collapse with occasional islets of hepatocytes and ductular proliferation with minimal elastosis to more advance lesions that can show nodular elastosis, dense fibrosis, thick blood vessels, and even thrombosed vessels. Generally, elastosis is composed of finely granular pale eosinophilic, amphophilic material that can be accompanied by fibrosis [4] Elastin is normally present in the portal tracts and large vessels wall. When there is an ischemic pattern of injury and in segmental atrophy, there is an increase in deposition of disorganized elastin within the liver parenchyma.

Singhi [2] first described this lesion in his study. The distinct histologic feature of this lesion is the elastosis. The limited elastosis in the early lesions makes the diagnosis more challenging. Collapsed hepatic parenchyma might not result in atrophy of the entire anatomical segment of liver but can be focal, affecting only few hepatic lobules.

The differential diagnosis for SAL is limited and includes hemangiomas, specifically sclerosing cavernous hemangiomas. Gonzalez et al. highlighted the utility of using elastin staining pattern to differentiate between sclerosing cavernous hemangiomas and SAL. [7] The presence of diffuse elastosis and large thick-walled vessels is more common in SAL. Careful attention for collapsed parenchyma with entrapped hepatocytes and ductular reaction is also a histologic clue for SAL.

None of the patients in our cohort had features of intrahepatic vascular disease, including focal nodular hyperplasia or porto-sinusoidal vascular disease. While most of the patients without a history of hypertension or cardiovascular disease had a history of malignancy, there was one patient with alcohol abuse disorder. Alcohol-related liver disease is known to potentially cause vascular injury in the liver [8]. However, the limited biopsy in this case did not show signs of alcohol-related liver injury or steatohepatitis. It is difficult to draw significant conclusions with regard to alcohol-related liver disease and SA, given the limited sampling of patients with alcohol abuse disorder in our cohort.

Radiologic features of SAL are variable and mimic metastasis or primary malignant lesions. These lesions are iso or hypoechoic on ultrasound with an ill-defined margin. On CT scan they are hypodense and non-enhancing and typically should lack fludeoxyglucose (FDG) uptake on positron emission tomography (PET) scan.

**Fig. 3:**
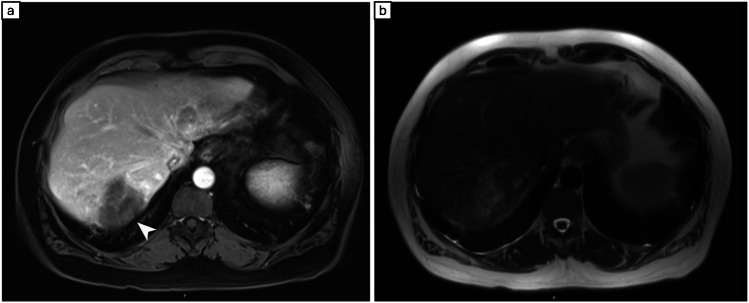
Irregular mass in hepatic segment 7 with hypointensity on T1 sequence (**a**) and central heterogeneous hyperintensity on T2-weighted images (**b**) with no evidence of restricted diffusion by magnetic resonance imaging (MRI)

In a study by Garg et al., most of the lesions were detected incidentally on CT scan. With added contrast, most lesions were hypodense [1] and one lesion was hyperdense compared to fatty liver parenchyma. On MRI, the lesions were iso to hyperintense on T2 and hypointense in T1 due to edema. In our study, unfortunately most of the lesions were not described by the radiologist, but among those available, hypodense lesions were the most commonly described on CT scan. The characteristic MRI features of segmental liver atrophy in one of our patients are illustrated and described in Fig. 3.

SAL in our study had almost equal distribution between men and woman, in contrast to studies [1, 2], which reported a female predominance.

Although these lesions are typically subcapsular based on the initial experience of Singhi et al. [2], in our cohort, lesions were widely distributed and equally distributed between left and right hepatic lobes. The gross images for none of our cases were available. Only 28% of the cases in our study showed subcapsular location. This was presumably a reflection of the large number of patients in our cohort with malignant neoplasms, which probably resulted in zones of vascular insufficiency in a wider range of locations.

The liver is a highly vascular organ and receives 20% of cardiac output. The hepatic blood flow is derived from the portal vein and hepatic artery providing 70% and 30% of the flow, respectively. Hepatic artery blood flow has a linear relation with blood pressure [9]. Previous studies on rats indicate that hypertension is a potential risk factor for hepatic fibrosis and liver injury through glucose intolerance and decreased IL-10-mediated or HO-1-induced anti-inflammatory mechanisms [10].

Segmental atrophy as the result of cardiovascular disease was previously suggested in an abstract form at the United States and Canadian Academy of Pathology (USCAP) conference in 2020 [3]. The reason that hepatic localized infarction might lead to formation of a mass lesion is not quite clear, but we hypothesize that the elastosis is a result of repair from the insulting ischemia and is itself tumefactive. Whether the ischemia is the result of a mass effect from an adjacent unsampled tumor or therapy-related changes or just simply from vascular disease, the final common pathway results in a mass-forming lesion. Most of our patient population had a history of cancer, chemotherapy, or radiation therapy. All these conditions presumably result in alterations of blood flow to the liver parenchyma. However, there was no evidence of metastasis or adjacent tumor in the biopsy or resection samples, and none of the patients had direct radiation therapy to the liver, which excludes the possibility of adjacent mass effect or radiation therapy effect on the liver parenchyma.

Collapsed hepatocytes interspersed with normal hepatocytes could be easily identified on reticulin stains, and predominant bile ductular reaction was highlighted on Cytokeratin 7 stain with mild to moderate elastotic changes highlighted on elastic stain. While clinicians and pathologists should always assume that liver segmental atrophy is associated with benign or malignant lesion of liver or biliary tract, they should keep in mind that cardiovascular disease by itself can be associated with the presence of this mass-like lesion on the imaging studies.

To the best of our knowledge, this study is the largest case series to date describing the histopathologic findings of liver segmental atrophy and the clinical correlation with cardiovascular disease and presumed vascular alterations reflective of treatment of neoplasms. However, this relationship does not imply a causal relationship, where cardiovascular disease directly influences the segmental atrophy of liver. Our study has several limitations inherent to the retrospective nature of study and especially lack of clinical information and radiologic findings on some of the consult cases. Another limitation of our study includes the older age of our patient cohort, which could lead to increased representation of cardiovascular disease.

In conclusion, this study highlights the histopathologic features of liver segmental atrophy and its association with cardiovascular disease or other causes of vascular perfusion impairment to the liver, whether benign or malignant. Pathologists should be familiar with the wide range of histologic features of SAL especially in the setting of a mass-forming lesion.

## Data Availability

Data from this study are available from the corresponding author upon reasonable request.
